# *TAF1* Transcripts and Neurofilament Light Chain as Biomarkers for X‐linked Dystonia‐Parkinsonism

**DOI:** 10.1002/mds.28305

**Published:** 2020-09-25

**Authors:** Jamal Al Ali, Christine A. Vaine, Shivangi Shah, Lindsey Campion, Ahmad Hakoum, Melanie L. Supnet, Patrick Acuña, Gabrielle Aldykiewicz, Trisha Multhaupt‐Buell, Niecy G.M. Ganza, John B.B. Lagarde, Jan K. De Guzman, Criscely Go, Benjamin Currall, Bianca Trombetta, Pia K. Webb, Michael Talkowski, Steven E. Arnold, Pike S. Cheah, Naoto Ito, Nutan Sharma, D. Cristopher Bragg, Laurie Ozelius, Xandra O. Breakefield

**Affiliations:** ^1^ Department of Neurology Massachusetts General Hospital and Harvard Medical School Boston Massachusetts USA; ^2^ Department of Neurology, The Collaborative Center for X‐linked Dystonia‐Parkinsonism Massachusetts General Hospital Charlestown Massachusetts USA; ^3^ Sunshine Care Foundation Roxas City Philippines; ^4^ Department of Neurology Jose R. Reyes Memorial Medical Center Metro Manila Philippines; ^5^ Center for Genomic Medicine, Mass General Research Institute Massachusetts General Hospital Boston Massachusetts USA; ^6^ Department of Neurology, Alzheimer's Clinical & Translational Research Unit Massachusetts General Hospital Charlestown Massachusetts USA; ^7^ Department of Human Anatomy Faculty of Medicine and Health Sciences, Universiti Putra Malaysia Serdang Malaysia; ^8^ Center for Molecular Imaging Research, Department of Radiology Massachusetts General Hospital Charlestown Massachusetts USA

**Keywords:** biomarkers, extracellular vesicles, neurofilament light chain, TAF1, XDP

## Abstract

**Background:**

X‐linked dystonia‐parkinsonism is a rare neurological disease endemic to the Philippines. Dystonic symptoms appear in males at the mean age of 40 years and progress to parkinsonism with degenerative pathology in the striatum. A retrotransposon inserted in intron 32 of the *TAF1* gene leads to alternative splicing in the region and a reduction of the full‐length mRNA transcript.

**Objectives:**

The objective of this study was to discover cell‐based and biofluid‐based biomarkers for X‐linked dystonia‐parkinsonism.

**Methods:**

RNA from patient‐derived neural progenitor cells and their secreted extracellular vesicles were used to screen for dysregulation of *TAF1* expression. Droplet‐digital polymerase chain reaction was used to quantify the expression of *TAF1* mRNA fragments 5′ and 3′ to the retrotransposon insertion and the disease‐specific splice variant TAF1‐32i in whole‐blood RNA. Plasma levels of neurofilament light chain were measured using single‐molecule array.

**Results:**

In neural progenitor cells and their extracellular vesicles, we confirmed that the *TAF1*‐3′/5′ ratio was lower in patient samples, whereas *TAF1*‐32i expression is higher relative to controls. In whole‐blood RNA, both TAF1‐3′/5′ ratio and TAF1‐32i expression can differentiate patient (n = 44) from control samples (n = 18) with high accuracy. Neurofilament light chain plasma levels were significantly elevated in patients (n = 43) compared with both carriers (n = 16) and controls (n = 21), with area under the curve of 0.79.

**Conclusions:**

*TAF1* dysregulation in blood serves as a disease‐specific biomarker that could be used as a readout for monitoring therapies targeting *TAF1* splicing. Neurofilament light chain could be used in monitoring neurodegeneration and disease progression in patients. © 2020 The Authors. *Movement Disorders* published by Wiley Periodicals LLC on behalf of International Parkinson and Movement Disorder Society.

X‐linked dystonia‐parkinsonism (XDP) is a rare neurological disease found predominantly in men with maternal ancestry linked to the island of Panay, Philippines. Dystonic symptoms typically appear in males at the mean age of 40 years and progress to parkinsonism with degenerative pathology in the striatum.^1^, ^2^, ^3^, ^4^, ^5^, ^6^, ^7^, ^8^, ^9^ In the most recent study in 2010, 505 patients with XDP were registered in the Philippines, 312 of which were survivors. The reported prevalence rate in the country overall was 0.31 per 100,000, and for Panay Island, 5.74 per 1,000,000.^2^ Recent efforts have narrowed down the causal mutation to a short interspersed elements (SINE) ‐ variable number of tandem repeats (VNTR) ‐ Alu (SVA) retrotransposon insertion in intron 32 of the *TAF1* gene^10^, ^11^, ^12^, ^13^ with an increased number of repeat elements in this SVA associated with earlier disease onset.^14^, ^15^ *TAF1* encodes the TATA‐box binding protein associated factor 1 (TAF1), the largest subunit of the multiprotein complex that makes up the general transcription factor complex, transcription factor II D (TFIID), which is critical to the formation of the RNA polymerase II preinitiation complex.^16^, ^17^, ^18^ The 38 constitutive exons of *TAF1* undergo various splicing events at the mRNA level, leading to differential expression of different isoforms combining exons 1‐38, or in some cases including alternative exons annotated as 32′, 34′, and 35′.^10^, ^11^, ^12^

In XDP cells, recent transcriptome assembly analysis showed that exon usage progressively decreases 3′ to intron 32.^10^ It is thought that the presence of the SVA in intron 32, possibly attributed to the formation of stacked guanine tetrads known as G4 motifs and/or reverse transcription of SVA‐derived sequences, is responsible for this transcriptional interference.^10^, ^14^ Recent studies have shown that the excision of the SVA rescues *TAF1* expression levels 3′ to the SVA.^10^, ^19^ Earlier studies compared *TAF1* expression in various tissues via reverse transcription polymerase chain reaction (RT‐qPCR) amplification using primers/probes that span the *TAF1* transcript, including sets that amplify 5′ to the SVA insertion (TAF1‐5′) and others 3′ to it (TAF1‐3′). Additional primers/probes were designed to amplify alternatively spliced isoforms, 1 of which is specific to a neuronal TAF1 isoform (nTAF1), which includes a 34′ exon.^11^ Consistent with the transcriptome assembly, TAF1‐3′ expression was lower than TAF1‐5′ expression in patient‐derived neuronal stem cells (NSCs)^10^ and primary fibroblasts^20^, ^21^ as well as RNA extracted from whole blood of patients with XDP compared with controls.^20^ The neuronal isoform, nTAF1, was shown to have lower expression in the brain of 1 patient with XDP^11^ and in patient‐derived NSCs^21^ based on RT‐qPCR amplification. In addition to decreased exon usage 3′ to intron 32, the presence of the SVA retrotransposon in *TAF1* induces partial retention of the proximal segment of intron 32 as well as multiple aberrant splicing events that terminate immediately proximal to the SVA insertion site, the most abundant of which was annotated as TAF1‐32i. This *TAF1* isoform is expressed at higher levels in XDP patient‐derived cell lines compared with controls, especially cells undergoing rapid division in the following rank order: induced pluripotent stem cells (iPSCs)>NSCs>fibroblasts>neurons.^10^ Clustered Regularly Interspaced Short Palindromic Repeats/CRISPR‐associated protein‐9 nuclease (CRISPR/Cas9) ‐based gene therapy on patient cell lines to selectively excise the SVA in intron 32 has normalized levels of TAF1‐32i.^10^

Considerable evidence supports neurofilament light chain (NfL) as a biomarker for neurodegeneration.^22^, ^23^, ^24^, ^25^, ^26^ Through the development of a highly sensitive fourth‐generation single molecule array, SiMoA (Quanterix Corporation, Billerica, MA), for detection in blood,^27^, ^28^ NfL has been reported in different neurodegenerative diseases as a blood‐based biomarker correlating with disease status, progression, and outcomes in different neurological diseases.^23^, ^28^ Specifically, it has been shown to be useful in the differential diagnoses of parkinsonian disorders.^24^, ^29^, ^30^

Clinically, XDP is diagnosed in patients with signs of dystonia and parkinsonism who have a positive history of affected relatives and maternal ancestry from Panay island.^2^ Cell‐based and biofluid‐based disease‐specific biomarkers will be needed to understand disease mechanisms, predict progression, and serve as noninvasive readouts for novel therapeutics. To identify a potential biomarker in XDP, we focused on RNA in extracellular vesicles (EVs). EVs are found within various biofluids, including blood, cerebrospinal fluid, and urine, and provide a protective environment for different RNA species, including mutant mRNAs, miRNAs, and splice variants.^31^, ^32^, ^33^, ^34^ In this study, we were able to quantitate levels of TAF1‐5′ and TAF1‐3′ as well as TAF1‐32i RNA expression in neural progenitor cells (NPCs), NPC‐derived EVs, and whole‐blood RNA from patients with XDP, female carriers, and healthy controls. We found that *TAF1* RNA species are differentially expressed among these study groups. In addition, we found that NfL is increased in the plasma of patients with XDP, adding XDP to the list of parkinsonian disorders and other neurodegenerative diseases with this biomarker feature. Overall, these studies implicate whole blood as a feasible biofluid to detect disease‐specific peripheral (and potentially brain derived) biomarkers in XDP, including differential *TAF1* transcript expression and increased NfL levels.

## 1 Methods

### 1.1 Participant Recruitment

Standardized tissue samples (blood and urine) and phenotype data (Table 1, Table S1, Table S2) were obtained from tissue and data banks, including the Dystonia Partners Research Bank approved by the Partners Human Research Committee (Boston, MA) and the XDP‐Partners Research Bank approved by Jose Reyes Memorial Medical Center (Manila, Philippines). All participants provided written informed consent for their samples and data to be used for genetic and cellular analyses. All participants were of Filipino decent.

**TABLE 1:** Participant characteristics

					*P* Value
	Patients with XDP	Female Carriers	Controls	Presymptomatic	
Plasma samples					
n	43	16	21	2	
Age, y (SD)	46.53 (9.37)	45.19 (24.04)	43.38 (14.46)	23.50 (7.78)	0.17
Sex, M/F	43/0	0/16	12/9	2/0	
Dystonia at presentation, n (%)	36 (83.72)	–	–	–	
Parkinsonism at presentation, n (%)	7 (16.27)	4 (25.00)	3 (13.28)	‐	
Whole‐blood RNA samples					
n	44	17	18	3	
Age, y (SD)	47.70 (10.55)	48.59 (24.19)	41.44 (14.63)	32.67 (16.80)	0.19
Sex, M/F	44/0	0/17	7/11	3/0	
Dystonia at presentation, n (%)	35 (79.54%)	–	–	–	
Parkinsonism at presentation, n (%)	9 (20.45%)	4 (23.53)	2 (11.11)	‐	

Plasma samples were used for plasma neurofilament light chain assays. Whole‐blood RNA samples were used for droplet‐digital polymerase chain reaction expression analysis of TAF1‐3′/5′ and TAF1‐32i. XDP, X‐linked dystonia‐parkinsonism; SD, standard deviation; M, male; F, female.

### 1.2 Sample Collection

To obtain plasma, whole blood from patients with XDP, female carriers, and controls was collected in 10 mL ethylenediaminetetraacetic acid tubes (BD Vacutainer Plastic Blood Collection Tubes with K2EDTA; ThermoFisher Scientific, Waltham, MA). Within 2 hours of collection, tubes were centrifuged at 1100*g* at room temperature for 10 minutes. Plasma was removed from the upper layer and then filtered through a Millex‐AA Syringe Filter Unit, 0.8 μm (MilliporeSigma, Burlington, MA), and aliquoted into cryovials and stored at −80°C. For blood RNA, whole blood from participants was collected using PAXgene tubes (Qiagen, Germantown, MD). Urine samples were collected using a sterile technique in 200 mL plastic bottles. Urine was centrifuged at low speed (2000*g*) with the supernatant passed through a Millex‐AA Syringe Filter Unit, 0.8 μm, and 50 mL aliquots were frozen at −80°C. Samples were shipped on ice or immediately processed whenever possible.

### 1.3 Cell Culture

Stable and highly expandable NPCs were derived from previously established iPSCs of patients with XDP and controls.^10^ NPC differentiation was performed based on a previously reported protocol,^35^ with several in‐house modifications. Sorting steps and subsequent culturing were followed as described in ref. 35 on Geltrex‐coated plates. NPCs at passage >9 were used for experiments. Subsequently, NPCs were grown for no more than 20 passages on Geltrex‐coated (ThermoFisher Scientific) tissue cultureware using Dulbecco's Modified Eagle Medium/Nutrient Mixture F‐12 (DMEM/F12 media) (ThermoFisher Scientific) supplemented with 2% B27 (ThermoFisher Scientific), 20 ng/mL of epidermal growth factor (EGF) (PeproTech, Rocky Hill, NJ), 20 ng/mL fibroblast growth factor (FGF) (MilliporeSigma), 5 ng/mL heparin (Sigma‐Aldrich), and 1% penicilin‐streptomycin (Corning, Corning, NY [catalog number 30‐002‐CI]).

### 1.4 EV Isolation from Culture Media, Plasma, and Urine Samples

Conditioned culture media from NPCs was collected after 24 to 48 hours of incubation. Using Amicon Ultra‐15 Centrifugal Filter Units, 100 kD (MilliporeSigma), 60 to 90 mL of the NPC‐conditioned media or 150 to 200 mL of the urine samples were concentrated to a final volume of 500 μL. The concentrated sample was then added to “qEV” columns (IZON Science, Medford, MA) for size exclusion chromatography to separate EVs from free protein according to size. Plasma samples were filtered through a 0.8 μm pore‐size membrane (Millex‐AA Syringe Filter Unit, MilliporeSigma) and then 500 μL were added directly to “qEV” columns for EV isolation. For all samples, the IZON “qEV” automatic fraction collector was used to collect fractions 7 to 11 containing EVs.^36^ Using Amicon Ultra‐0.5 Centrifugal Filter Units, 30 kD (MilliporeSigma), a total of 2.5 mL EV‐enriched filtrate was then concentrated to 100 to 200 μL for downstream RNA isolation.

### 1.5 RNA Extraction

RNA was extracted from NPC cell pellets and EV concentrate from NPC, plasma, and urine by adding QIAzol (Qiagen); samples were then mixed with one‐fifth volume of chloroform with brief centrifugation at 12,000*g* to allow for phase separation. The aqueous phase was processed using miRNeasy spin columns (Qiagen) with on‐column DNase digestion as recommended. RNA was extracted from whole blood using the PAXgene Blood RNA Kit according to the manufacturer's protocol (Qiagen). The resulting RNA samples were quantified by Nanodrop (ThermoFischer Scientific) and Bioanalyzer 2100 (Agilent Technologies, Santa Clara, CA). RNA from NPCs (40 ng), NPC EVs (2–10 ng), plasma EVs (0.5–3.5 ng), urine EVs (0.5–3.5 ng), and whole blood (40 ng) were reverse transcribed using the SuperScript VILO cDNA Synthesis Kit (ThermoFisher Scientific).

### 1.6 **RT‐qPCR**

**FIG. 1:**
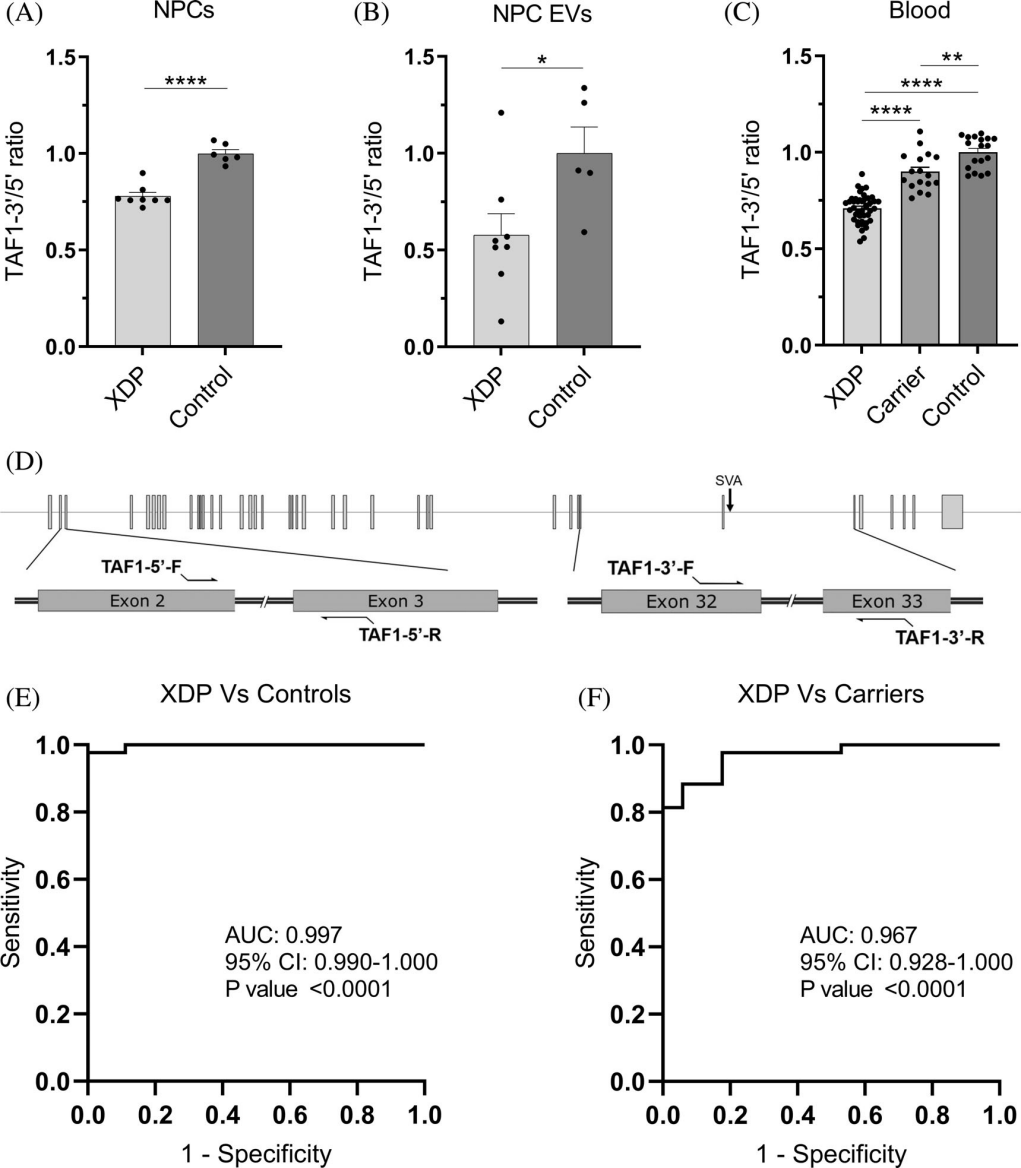
*TAF1*‐3′/5′ ratio expression in NPCs, NPC EVs, and peripheral blood. (**A**) RT‐qPCR expression of TAF1‐3′/5′ ratio in XDP (n = 8) and control (n = 6) NPCs. (**B**) Droplet‐digital polymerase chain reaction expression of TAF1‐3′/5′ ratio in XDP (n = 8) and control (n = 5) EVs isolated from NPCs in culture. (**C**) Droplet‐digital polymerase chain reaction expression of TAF1‐3′/5′ ratio in patients with XDP (n = 43), female carriers (n = 17), and controls (n = 18) in whole‐blood RNA. Each dot represents a unique sample. Mean and standard error are represented. (**D**) Schematic for TAF1‐5′ primers relative to TAF1 exons 2 and 3 and TAF1‐3′ primers relative to the SVA and TAF1 exons 32 and 33. (**E**) Receiver operating characteristic analysis of TAF1‐3′/5′ ratio in whole‐blood RNA in XDP versus control samples. (**F**) Receiver operating characteristic analysis of TAF1‐3′/5′ ratio in whole‐blood RNA in XDP versus female carrier samples. AUC, area under the curve; CI, confidence interval; EV, extracellular vesicle; NPCs, neural progenitor cells; XDP, X‐linked dystonia‐parkinsonism. *P < 0.05, **P < 0.01, ****P < 0.0001.

**FIG. 2:**
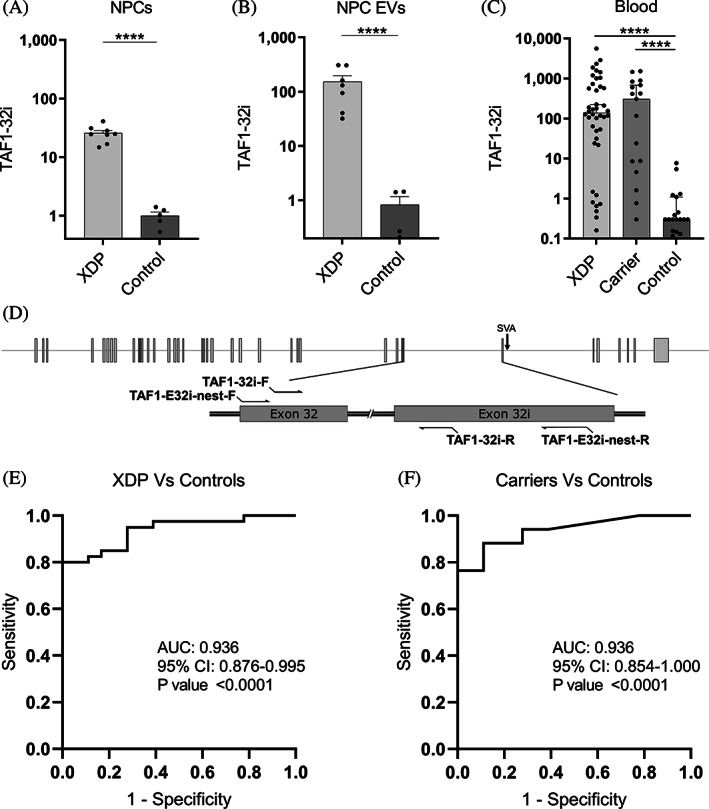
TAF1‐32i expression in NPCs, NPC EVs, and peripheral blood. (**A**) RT‐PCR expression of TAF1‐32i in XDP (n = 8) and control (n = 5) NPCs. (**B**) Droplet‐digital polymerase chain reaction expression of TAF1‐32i in XDP (n = 8) and control (n = 5) EVs isolated from NPCs in culture. (**C**) Droplet‐digital polymerase chain reaction expression of TAF1‐32i ratio in XDP (n = 40), female carriers (n = 17), and controls (n = 18) in whole‐blood RNA. Dotted line shown at y = 5 to separate background expression seen in all 17 controls, in 8 XDP samples, and 3 carrier samples (y < 5). Each dot represents a unique sample. Means with standard errors are represented. Data are logarithmically transformed. (**D**) Schematic for TAF1‐32i and TAF1‐32i preamplification primers (TAF1‐E32i‐nest) relative to the SVA and TAF1 exons 32 and 32i. (**E**) receiver operating characteristic analysis of TAF1‐32i expression in whole‐blood RNA in XDP versus control samples. (**F**) Receiver operating characteristic analysis of TAF1‐32i expression in whole‐blood RNA in female carrier versus control samples. AUC, area under the curve; CI, confidence interval; EV, extracellular vesicle; NPCs, neural progenitor cells; XDP, X‐linked dystonia‐parkinsonism. ****P < 0.0001.

RT‐qPCR was performed using custom TAF1 TaqMan primers (ThermoFisher Scientific) as previously described^11^ to detect TAF1‐5′ (forward 5′ ➔ 3′: GACTGACGGTGCCTTGGT, reverse 5′ ➔ 3′: GTCTGAATAGTCCACAGCATCTTCT, reporter probe 5′ ➔ 3′: ACCCACCCTTCATCATTT, amplicon size 70 bp) and TAF1‐3′ (forward 5′ ➔ 3′: ACCTTATTCTGGCCAACAGTGTT, reverse 5′ ➔ 3′: ACAATCTCCTGGGCAGTCTTAGTAT, reporter probe 5′ ➔ 3′: ACTCTCAGGTCCATTATAC, amplicon size 73 bp) (Fig. 1D). The TAF1‐32i Taqman primer set (TaqMan Custom Assay identification number: AJWR28J; ThermoFischer Scientific) was used to detect the presence of the exon 32/intron 32 splice site such that the forward primer bound to exon 32 and the reverse primer bound to the spliced region of intron 32, named here as exon 32i (Fig. 2D), and the probe spanned the splice junction.^10^

### 1.7 PCR Preamplification

TAF1‐E32i‐preamplification primers (forward 5′ ➔ 3′: ACATCTCCAAGCACAAGTATCA) and (reverse 5′ ➔ 3′: GTAATGTACCAATATAAATTTCCTGGTTT, amplicon size 208 bp) were used to amplify a region of 208 bp spanning the TAF1‐32i splice variant site (Fig. 2D). Reverse‐transcribed cDNA was used as a template to run 10 to 15 cycles of PCR with Phusion Hot Start Flex DNA Polymerase according to the manufacturer's protocol (New England Biolabs, Ipswich, MA). The amplified cDNA mixture was then cleaned and concentrated using DNA Clean and Concentrator‐5 Kit (Zymo Research, Irvine, CA) according to the manufacturer's protocol.

### 1.8 Droplet‐Digital PCR

Gene expression was analyzed via droplet‐digital PCR using the same Taqman probes used for RT‐qPCR. Using the protocol listed by the manufacturer, the droplets were generated with the DG32 Cartridge using the Automated Droplet Generator QX200 AutoDG Droplet Digital PCR System from Bio‐Rad (Hercules, CA), and PCR was performed with thermal cycling conditions as described by the manufacturer. QX200 Droplet Reader and QuantaSoft Software (Bio‐Rad) were used to analyze gene expression.

### 1.9 **NfL SiMoA**

NfL concentrations were measured using the HD‐X NfL kit (catalog number 103186) on the SiMoA HD‐X Analyzer (Quanterix Corporation). Plasma samples were centrifuged at 10,000*g* for 10 minutes and diluted 1:4 in sample buffer (100 μL plasma). All samples were run in duplicates. The assay was run according to manufacturer's protocol. This assay has a lower limit of quantification of 0.174 pg/mL, a limit of detection of 0.038 pg/mL (range 0.003–0.079 pg/mL), and a dynamic range in serum/plasma of 0 to 1800 pg/mL.

### 1.10 Statistical Analysis

**FIG. 3:**
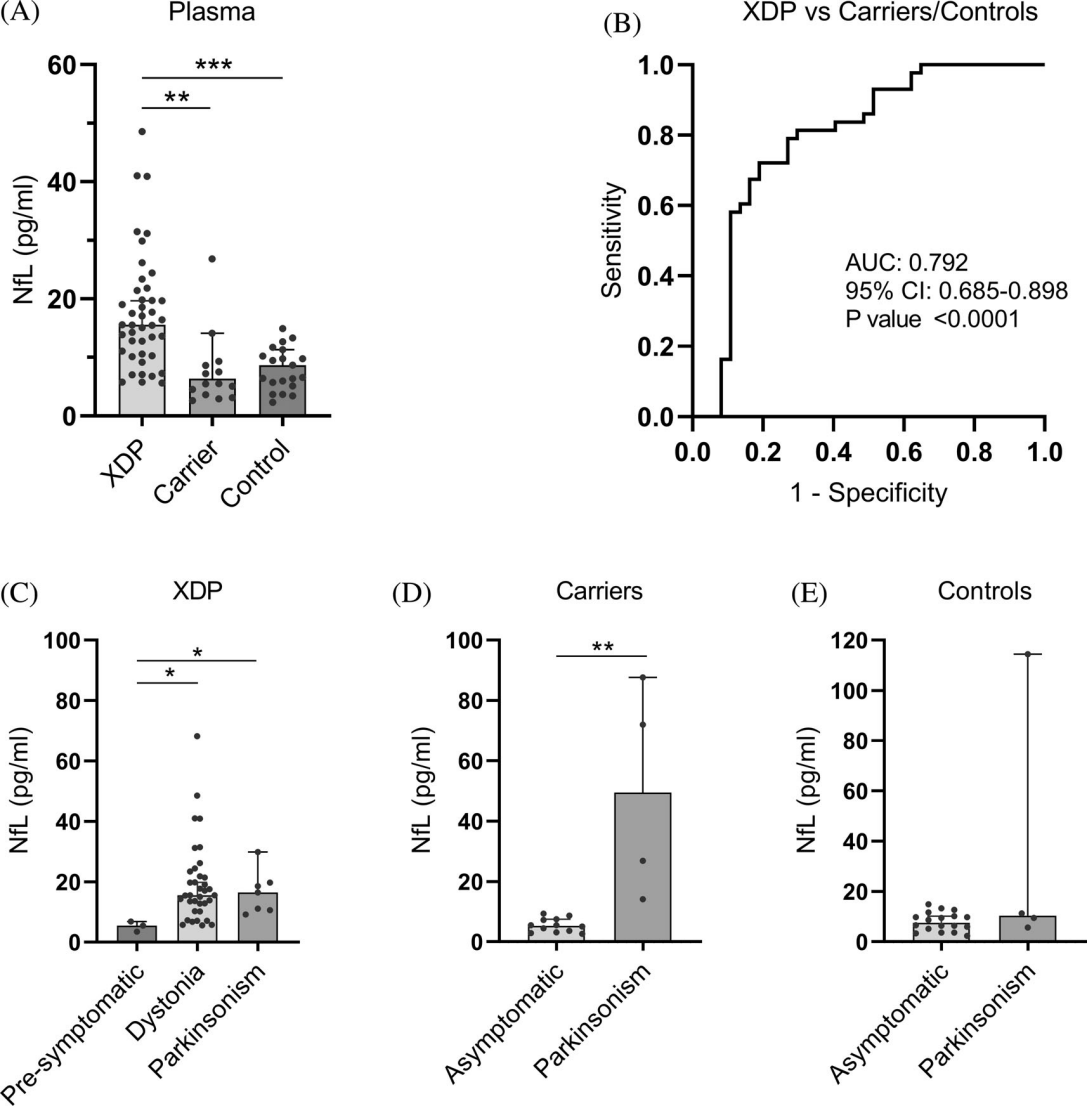
NfL in plasma. (**A**) NfL protein concentration (pg/ml) in plasma from patients with XDP (n = 43), female carriers (n = 16), and healthy controls (n = 21) using SiMoA. Medians with 95% CIs are represented. (**B**) Receiver operating characteristic analysis of plasma NfL in patients with XDP versus female carriers and controls. (**C**) Subgroup analysis of plasma NfL in patients with XDP according to the type of symptom, if any, at disease onset. (**D**) Subgroup analysis of plasma NfL in female carriers according to the type of symptom at presentation, if any. (**E**) Subgroup analysis of plasma NfL in controls according to the type of symptom at presentation, if any. Medians with 95% CIs represented. AUC, area under the curve; CI, confidence interval; NfL, neurofilament light chain; XDP, X‐linked dystonia‐parkinsonism. *P < 0.05, **P < 0.01, ***P < 0.001.

GraphPad Prism version 8.0.0 (GraphPad Software, San Diego, CA) was used to analyze RNA expression, NfL plasma quantification, receiver operating characteristic curves, and linear regression analysis. The Student *t* test was used to compare 2 means from data sets that exhibited normal distribution (Figs. 1A,B and 2A,B). One‐way analysis of variance was used to compare 3 or more means for normally distributed data (Fig. 1C). Data from TAF1‐32i expression (Fig. 2) were logarithmically transformed. The Kruskal‐Wallis test was used to compare 3 or more medians from data sets that were not normally distributed (Figs. 2C, 3A,C,D,E). Statistical significance was considered for *P* value <0.05.

## 2 Results

### 2.1 TAF1 Expression in NPCs, NPC EVs, and Peripheral Blood

To accurately compare *TAF1* RNA dysregulation in XDP using a variety of sources (cell lysates, EVs, and whole blood), we evaluated *TAF1* transcript expression as a ratio of TAF1‐3′/5′ using TAF1‐5′ expression for normalization, as the expression of classic housekeeping genes was found to vary greatly among tissues. In our NPC cell model, TAF1‐5′ expression showed no significant difference between XDP and control lines when corrected to the housekeeping gene *GAPDH*, whereas TAF1‐3′ expression was significantly lower (data not shown) in XDP samples, consistent with decreased *TAF1* transcription expression 3′ to intron 32 in other cell types including iPSCs, NSCs, and fibroblasts.^10^, ^20^, ^21^ We confirmed that TAF1‐3′/5′ ratio reflects the expected dysregulation in NPCs, with 22% lower expression (*P* < 0.0001) in XDP NPCs (n = 8) relative to controls (n = 6) (Fig. 1A). In NPC EVs, TAF1‐3′/5′ expression was 42% lower (*P* < 0.05) in XDP samples (n = 8) compared with controls (n = 5) (Fig. 1B). In peripheral blood, XDP samples (n = 3) showed a 29% decrease (*P* < 0.0001) in TAF1‐3′/5′ ratio relative to controls (n = 18), and its expression in female carriers (n = 17) was on average 19% higher (*P* < 0.0001) than patient samples and only 10% lower (*P* < 0.01) than controls (Fig. 1C). TAF1‐3′/5′ expression can accurately differentiate patients with XDP from control samples (area under the curve (AUC) = 0.997, *P* < 0.0001) (Fig. 1E) and patients with XDP from carrier samples (AUC = 0.967, *P* < 0.0001) (Fig. 1F). As for the 3 males with presymptomatic XDP, their TAF1‐3′/5′ ratios were 25%, 26%, and 19% lower than the average of the control samples (Table S1). Linear regression analysis between TAF1‐3′/5′ expression and subject parameters, including, age, age at disease onset, duration of disease, repeat size, and symptoms, were not revealing (data not shown).

### 2.2 TAF1‐32i Expression in NPCs, NPC EVs, and Peripheral Blood

qPCR analysis of the TAF1‐32i splice variant in NPCs derived from iPSCs showed on average a 25‐fold increase (*P* < 0.0001) in levels in XDP cells (n = 8) relative to controls (n = 6) (Fig. 2A). These data support a similar expression signature shown previously in other XDP‐derived cell models, including fibroblasts, iPSCs, and NSCs.^10^ To increase our detection limit of this lowly expressed splice variant, we performed preamplification of the region in cDNA via PCR then used droplet‐digital PCR for quantitative detection using TaqMan primer/probes. Using this method, we found that the TAF1‐32i splice variant is abundant in EVs derived from XDP cell lines (n = 8), with a 184‐fold increase (*P* < 0.0001) in expression relative to EVs derived from control NPCs (n = 5) (Fig. 2B).

Next, we quantified TAF1‐32i expression in whole‐blood RNA from PAX tubes from patients with XDP (n = 40), female carriers (n = 17), and controls (n = 18) (Tables S1 and S2) using the same preamplification methods as previously. We found high expression levels of the TAF1‐32i splice variant in peripheral blood from the patients with XDP (*P* < 0.0001) and female carriers (*P* < 0.0001) compared with controls (Fig. 2C). Similar to the TAF1‐3′/5′ ratio, TAF1‐32i expression can accurately differentiate patients with XDP from control samples (AUC = 0.939, *P* < 0.0001) (Fig. 2E) and female carriers from control samples (AUC = 0.936, *P* < 0.0001) (Fig. 2E). Two males with presymptomatic XDP had TAF1‐32i expression 24 and 2300 times higher than the average of the control samples (Table S1).Thus, for the first time, we demonstrated evidence of high levels of the aberrant splice variant TAF1‐32i in peripheral blood samples in patients with XDP and female carriers. Linear regression analysis between TAF1‐32i expression and subject parameters were not revealing (data not shown).

### 2.3 NfL in XDP Plasma

Using the SiMoA platform, we were able to assay NfL protein levels in plasma from patients with XDP (n = 43), female carriers (n = 16), and healthy controls (n = 21) (Table 1). We detected abnormally high levels of plasma NfL in patients with XDP with a median of 15.57 pg/mL compared with 6.38 pg/mL in female carriers (*P* < 0.001) and 8.66 pg/mL in healthy controls (*P* < 0.01) (Fig. 3A). Plasma NfL is capable of differentiating samples from patients with XDP from female carriers and control samples together with a high AUC of 0.792 (*P* < 0.0001) (Fig. 3B). Three males with presymptomatic XDP aged between 40 and 51 years showed low levels of plasma NfL at a median of 5.49 pg/mL versus a median of 15.54 (*P* < 0.05) and 16.42 pg/mL (*P* < 0.05) in patients with XDP with dystonia and parkinsonism symptoms at disease onset, respectively (Fig. 3C). Four female carriers, aged between 77 and 84 years, had parkinsonism symptoms, but no specific clinical diagnosis. Their NfL levels were high at a median of 49.42 pg/μL versus 5.26 pg/μL in asymptomatic female carriers (*P* < 0.01) (Fig. 3D). Four male controls, aged between 32 and 53, also had parkinsonism symptoms with no specific clinical diagnosis, but did not carry the XDP‐specific SVA. Unlike the female carriers, only 1 symptomatic control had high NfL levels, and the median level (9.47 pg/mL) was not significantly higher than asymptomatic controls (8.66 pg/mL) (Fig. 3E). Linear regression analysis between plasma NfL levels and subject parameters were not revealing (Fig. S1).

## 3 Discussion

Our results confirm transcriptional dysregulation of *TAF1* in our NPC cell model, in NPC‐secreted EVs, and in peripheral blood in patients with XDP and female carriers compared with neurologically healthy controls. Dysregulation manifested as a decreased ratio of TAF1‐3′/5’ mRNA and increased expression of the aberrant splice variant TAF1‐32i. This expression signature for *TAF1* transcripts can be used as an XDP‐specific biomarker. In addition, we showed evidence of increased NfL protein levels in XDP plasma, which can be used as a nonspecific disease biomarker of neurodegeneration.

Previous work on *TAF1* transcripts has consistently shown decreased exon expression 3′ to the SVA insertion in intron 32.^10^, ^11^, ^20^, ^21^ Most work was done on XDP patient‐derived cell lines, including fibroblasts,^10^, ^20^ iPSCs,^10^ and NSCs.^10^, ^21^ In addition, TAF1‐32i splice variant expression has been quantified in the same patient cell lines and was directly linked to the presence of the SVA in intron 32.^10^ As expected, our NPC cells derived from patients with XDP showed similar *TAF1* dysregulation, reflected by the decreased ratio of TAF1‐3′/5′ transcripts and the increased expression of the aberrant splice variant TAF1‐32i. Furthermore, we showed that *TAF1* fragments, including 5′, 3′, and 32i, are secreted in EVs isolated from the NPC culture media and that the expression of these fragments in EVs reflected the dysregulation occurring in XDP NPC lines. These findings shed light on potential cellular processing mechanisms for *TAF1* RNA in XDP. Cells could be using EVs to actively discard the aberrant splice variant TAF1‐32i.^37^, ^38^, ^39^, ^40^ Alternatively, cells may indiscriminately package RNA into EVs,^37^, ^38^, ^40^, ^41^ which would explain why NPC EVs reflect the *TAF1* dysregulation seen in the source cell. It is important to note that the expression of the TAF1‐3′/5′ ratio in NPC EVs is highly variable relative to its expression in NPCs. This could be attributed to the extensive sample handling necessary for our analysis or to an underlying biological process pertaining to the cellular handling of different *TAF1* fragments as mentioned previously. Irrespective of the secretion mechanism, EVs have been implicated in the pathogenesis of neurological disorders^42^, ^43^, ^44^ and can be used as disease biomarkers when isolated from biofluids.^33^, ^45^, ^46^, ^47^, ^48^, ^49^, ^50^ We assayed extracellular RNA from plasma and urine EVs from patient and control samples using size exclusion chromatography.^36^ Our low RNA yields (0.5–3.5 ng total per sample) did not allow us to amplify TAF1‐32i in any of these samples (data not shown). With the improving techniques in isolating and assaying EV contents, our findings indicate that EVs from biofluids may be useful in assaying *TAF1* transcripts and splice variants from patient samples.

We also interrogated *TAF1* dysregulation in whole‐blood RNA. A previous study showed a reduction in TAF1‐3′ expression in XDP whole‐blood RNA compared with TAF1‐5′, which remained unchanged relative to control samples.^20^ Our results support those findings by showing that the TAF1‐3′/5′ ratio is effective in distinguishing patients with XDP, asymptomatic female carriers, and healthy controls. Interestingly, 3 presymptomatic males with the XDP haplotype, aged 18, 29, and 51, had TAF1‐3′/5′ levels similar to patients with XDP. We hypothesize that this marginal yet consistent decrease in TAF1‐3′/5′ may correlate with disease status and could be related to an active underlying pathological process in both presymptomatic and symptomatic patients with XDP. TAF1‐32i XDP‐specific splice variant was discovered by transcriptome assembly and shown to be expressed in dividing cells, including iPSC, NSCs, and fibroblasts.^10^ Ours is the first study to show evidence of TAF1‐32i expression in whole‐blood RNA, shedding light on a systemic dysregulation in *TAF1* expression and the potential use of *TAF1* RNA fragments in biofluids as an XDP disease‐specific biomarker.

*TAF1* expression in asymptomatic female carriers had not been studied before, and these are the first experiments to show *TAF1* dysregulation in whole‐blood RNA from carriers. Most female carriers of the disease‐causing haplotype are asymptomatic, with only 14 symptomatic cases reported to date of more than 500 male patients.^51^, ^52^, ^53^, ^54^ Although skewed X‐chromosome inactivation has been reported as the underlying mechanism in at least 1 case of an XDP‐carrier symptomatic female,^52^ it has also been shown to be the underlying protective mechanism against X‐linked neurological diseases caused by *TAF1* coding mutations.^55^, ^56^, ^57^ We hypothesize that skewed X‐chromosome inactivation may attenuate *TAF1* dysregulation, which in turn protects female carriers from the downstream effects of a significant decrease in normal *TAF1* expression.

To the best of our knowledge, there are no published studies measuring plasma proteins in XDP. We used SiMoA technology to assay NfL, a nonspecific marker of neurodegeneration,^23^, ^25^, ^28^ in the plasma of patients with XDP, males with presymptomatic XDP, female carriers, and healthy controls. We show for the first time evidence of abnormally high levels of NfL in XDP plasma, reflecting a neurodegenerative process occurring in the brains of patients with XDP, but not in males with presymptomatic XDP or asymptomatic female carriers. Our results are consistent with the knowledge that XDP is a disease of basal ganglia neurodegeneration.^1^, ^2^, ^3^, ^4^, ^9^ This adds XDP to the list of atypical parkinsonian syndromes with increased plasma NfL.^24^, ^29^, ^30^

Although NfL may be a nonspecific biomarker of neurodegeneration in XDP, it may still be useful in monitoring disease onset and progression in presymptomatic and symptomatic patients under therapeutic treatment. *TAF1* dysregulation acts as a disease‐specific biomarker that makes it an attractive readout for target engagement by new therapies, particularly those targeting *TAF1* expression and splicing regulation. Analyzed together, these nonspecific and specific biomarkers may reflect disease progression and serve as robust readouts for targeted therapeutics. Future longitudinal studies are needed to follow cohorts of XDP presymptomatic and symptomatic patients and female carriers to better detect clinical correlations between our developed biomarkers and different disease parameters. Such studies are also key in determining the usefulness of NfL in detecting evidence of neurodegeneration and disease activity in males and females with presymptomatic XDP.

## Author Roles

(1) Research Project: A. Conception, B. Organization, C. Execution; (2) Statistical Analysis: A. Design, B. Execution, C. Review and Critique; (3) Manuscript: A. Writing of the First Draft, B. Review and Critique.

J.A.A.: 1A, 1B, 1C, 2A, 2B, 2C, 3A, 3B

C.A.V.: 1A, 1B, 1C,2C, 3A, 3B

S.S.: 1A, 1B, 1C, 2C, 3B

L.C.: 1A, 1B, 1C, 2C, 3B

A.H.: 1A, 1B, 1C, 2C, 3B

M.L.S.: 1A, 1B, 1C, 2C, 3B

P.A.: 1A, 1B, 1C, 2C, 3B

G.A.: 1A, 1B, 1C, 2C, 3B

T.M.‐B.: 1A, 1B, 1C, 2C, 3B

N.G.M.G.: 1B, 1C, 2C, 3B

J.B.B.L.: 1B, 1C, 2C, 3B

J.K.D.G.: 1B, 1C, 2C, 3B

C.G.: 1B, 1C, 2C, 3B

B.C.: 1A, 1C, 2C, 3B

B.T.: 1A, 1C, 2C, 3B

P.K.W.: 1A, 1C, 2C, 3B

M.T.: 1A, 1B, 2C, 3B

S.E.A.: 1A, 1B, 2C, 3B

P.S.C.: 1A, 1B, 2C, 3B

N.I.: 1A, 1B, 2B, 3A, 3B

N.S.: 1A, 1B, 2B, 3A, 3B

D.C.B.: 1A, 1B, 2B, 3A, 3B

L.O.: 1A, 1B, 2B, 3A, 3B

X.O.B.: 1A, 1B, 2A, 2B, 3A, 3B

## Full financial disclosures for the previous 12 months:

N.S. reports grants from the National Institutes of Health and funding from the Massachusetts General Hospital Collaborative Center for X‐Linked Dystonia‐Parkinsonism. D.C.B. reports grants from the National Institutes of Health and funding from the Massachusetts General Hospital Collaborative Center for X‐Linked Dystonia‐Parkinsonism. L.O. reports grants from the National Institutes of Health and funding from the Massachusetts General Hospital Collaborative Center for X‐Linked Dystonia‐Parkinsonism. X.O.B. reports grants from the National Institutes of Health and funding from the Massachusetts General Hospital Collaborative Center for X‐Linked Dystonia‐Parkinsonism. All other authors have nothing to disclose.

## Supporting information


**Figure S1.** Correlation analysis between neurofilament light chain (NfL) in plasma and disease parameters. (A) Linear correlation between plasma NfL and age in patients with X‐linked dystonia‐parkinsonism (XDP). (B) Linear correlation between plasma NfL and age in asymptomatic female carriers. (C) Linear correlation between plasma NfL and age in asymptomatic controls. (D) Linear correlation between plasma NfL and age at onset of disease in patients with XDP. (E) Linear correlation between plasma NfL and duration of disease in patients with XDP. (F) Linear correlation between plasma NfL and hexanucleotide repeat size in patients with XDP. (G) Linear correlation between plasma age at onset of disease and hexanucleotide repeat size in patients with XDP.

**Table S1.** Patient characteristics and results.

**Table S2.** Carrier and control characteristics and results.

## References

[1] Hanssen H , Heldmann M , Prasuhn J , et al. Basal ganglia and cerebellar pathology in X‐linked dystonia‐parkinsonism. Brain 2018;141:2995–3008. 30169601 10.1093/brain/awy222

[2] Lee LV , Rivera C , Teleg RA , et al. The unique phenomenology of sex‐linked dystonia parkinsonism (XDP, DYT3, “Lubag”). Int J Neurosci. 2011;121(suppl):3–11. 10.3109/00207454.2010.526728 21047175

[3] Goto S , Lee LV , Munoz EL , et al. Functional anatomy of the basal ganglia in X‐linked recessive dystonia‐parkinsonism. Ann Neurol 2005;58:7–17. 15912496 10.1002/ana.20513

[4] Hanssen H , Prasuhn J , Heldmann M , et al. Imaging gradual neurodegeneration in a basal ganglia model disease. Ann Neurol 2019;86:517–526. 31376168 10.1002/ana.25566

[5] Lee LV , Maranon E , Demaisip C , et al. The natural history of sex‐linked recessive dystonia parkinsonism of Panay, Philippines (XDP). Parkinsonism Relat Disord 2002;9:29–38. 12217620 10.1016/s1353-8020(02)00042-1

[6] Lee LV , Kupke KG , Caballar‐Gonzaga F , Hebron‐Ortiz M , Müller U . The phenotype of the X‐linked dystonia‐parkinsonism syndrome: an assessment of 42 cases in The Philippines. Med (United States) 1991;70:179–187. 10.1097/00005792-199105000-00002 2030641

[7] Goto S , Kawarai T , Morigaki R , et al. Defects in the striatal neuropeptide Y system in X‐linked dystonia‐parkinsonism. A J Neurol 2013;136(5):1555–1567. 10.1093/brain/awt084 23599389

[8] Evidente VGH , Advincula J , Esteban R , et al. Phenomenology of “Lubag” or X‐linked dystonia‐parkinsonism. Mov Disord 2002;17:1271–1277. 12465067 10.1002/mds.10271

[9] Bruggemann N , Rosales RL , Waugh JL , et al. Striatal dysfunction in X‐linked dystonia‐parkinsonism is associated with disease progression. Eur J Neurol 2017;24:680–686. 28236370 10.1111/ene.13256

[10] Aneichyk T , Hendriks WT , Yadav R , et al. Dissecting the causal mechanism of X‐linked dystonia‐parkinsonism by integrating genome and transcriptome assembly. Cell 2018;172:897–909.e21. 29474918 10.1016/j.cell.2018.02.011 PMC5831509

[11] Makino S , Kaji R , Ando S , et al. Reduced neuron‐specific expression of the TAF1 gene is associated with X‐linked dystonia‐parkinsonism. Am J Hum Genet 2007;80:393–406. 17273961 10.1086/512129 PMC1821114

[12] Herzfeld T , Nolte D , Muller U . Structural and functional analysis of the human TAF1/DYT3 multiple transcript system. Mamm Genome. 2007;18:787–795. 17952504 10.1007/s00335-007-9063-z

[13] Nolte D , Niemann S , Müller U . Specific sequence changes in multiple transcript system DYT3 are associated with X‐linked dystonia parkinsonism. Proc Natl Acad Sci U S A 2003;100:10347–10352. 12928496 10.1073/pnas.1831949100 PMC193564

[14] Bragg DC , Mangkalaphiban K , Vaine CA , et al. Disease onset in X‐linked dystonia‐parkinsonism correlates with expansion of a hexameric repeat within an SVA retrotransposon in TAF1. Proc Natl Acad Sci U S A 2017;114:E11020–E11028. 29229810 10.1073/pnas.1712526114 PMC5754783

[15] Westenberger A , Reyes CJ , Saranza G , et al. A hexanucleotide repeat modifies expressivity of X‐linked dystonia parkinsonism. Ann Neurol 2019;85:812–822. 30973967 10.1002/ana.25488

[16] Thomas MC , Chiang CM . The general transcription machinery and general cofactors. Crit Rev Biochem Mol Biol 2006;41:105–178. 16858867 10.1080/10409230600648736

[17] Louder RK , He Y , López‐Blanco JR , Fang J , Chacón P , Nogales E . Structure of promoter‐bound TFIID and model of human pre‐initiation complex assembly. Nature 2016;531:604–609. 27007846 10.1038/nature17394 PMC4856295

[18] Anandapadamanaban M , Andresen C , Helander S , et al. High‐resolution structure of TBP with TAF1 reveals anssschoring patterns in transcriptional regulation. Nat Struct Mol Biol 2013;20:1008–1014. 23851461 10.1038/nsmb.2611 PMC4972576

[19] Rakovic A , Domingo A , Grütz K , et al. Genome editing in induced pluripotent stem cells rescues *TAF1* levels in X‐linked dystonia‐parkinsonism. Mov Disord 2018;33:1108–1118. 30153385 10.1002/mds.27441

[20] Domingo A , Amar D , Grutz K , et al. Evidence of TAF1 dysfunction in peripheral models of X‐linked dystonia‐parkinsonism. Cell Mol Life Sci 2016;73:3205–3215. 26879577 10.1007/s00018-016-2159-4 PMC11108471

[21] Ito N , Hendriks WT , Dhakal J , et al. Decreased N‐TAF1 expression in X‐linked dystonia‐parkinsonism patient‐specific neural stem cells. Dis Model Mech 2016;9:451–462. 26769797 10.1242/dmm.022590 PMC4852502

[22] Gaetani L , Blennow K , Calabresi P , Di Filippo M , Parnetti L , Zetterberg H . Neurofilament light chain as a biomarker in neurological disorders. J Neurol Neurosurg Psychiatry 2019;90:870–881. 30967444 10.1136/jnnp-2018-320106

[23] Benedet AL , Ashton NJ , Pascoal TA , et al. Plasma neurofilament light associates with Alzheimer's disease metabolic decline in amyloid‐positive individuals. Alzheimers Dement (Amst) 2019;11:679–689. 31673598 10.1016/j.dadm.2019.08.002 PMC6816316

[24] Hansson O , Janelidze S , Hall S , et al. Blood‐based NfL: a biomarker for differential diagnosis of parkinsonian disorder. Neurology 2017;88:930–937. 28179466 10.1212/WNL.0000000000003680 PMC5333515

[25] Petzold A . Neurofilament phosphoforms: surrogate markers for axonal injury, degeneration and loss. J Neurol Sci 2005;233:183–198. 15896809 10.1016/j.jns.2005.03.015

[26] Quiroz YT , Zetterberg H , Reiman EM , et al. Plasma neurofilament light chain in the presenilin 1 E280A autosomal dominant Alzheimer's disease kindred: a cross‐sectional and longitudinal cohort study. Lancet Neurol 2020;19:513–521. 32470423 10.1016/S1474-4422(20)30137-X PMC7417082

[27] Kuhle J , Barro C , Andreasson U , et al. Comparison of three analytical platforms for quantification of the neurofilament light chain in blood samples: ELISA, electrochemiluminescence immunoassay and Simoa. Clin Chem Lab Med 2016;54:1655–1661. 27071153 10.1515/cclm-2015-1195

[28] Khalil M , Teunissen CE , Otto M , et al. Neurofilaments as biomarkers in neurological disorders. Nat Rev Neurol 2018;14:577–589. 30171200 10.1038/s41582-018-0058-z

[29] O'Bryant SE , Edwards M , Zhang F , et al. Potential two‐step proteomic signature for Parkinson's disease: pilot analysis in the Harvard Biomarkers Study. Alzheimer's Dement 2019;11:374–382. 10.1016/j.dadm.2019.03.001 PMC6502745 31080873

[30] Archer DB , Mitchell T , Burciu RG , et al. Magnetic resonance imaging and neurofilament light in the differentiation of parkinsonism. Mov Disord 2020;35:1388–1395. 32357259 10.1002/mds.28060 PMC8316785

[31] Skog J , Würdinger T , van Rijn S , et al. Glioblastoma microvesicles transport RNA and proteins that promote tumour growth and provide diagnostic biomarkers. Nat Cell Biol 2008;10:1470–1476. 19011622 10.1038/ncb1800 PMC3423894

[32] Burgos K , Malenica I , Metpally R , et al. Profiles of extracellular miRNA in cerebrospinal fluid and serum from patients with Alzheimer's and Parkinson's diseases correlate with disease status and features of pathology. PLoS ONE 2014;9:e94839. 24797360 10.1371/journal.pone.0094839 PMC4010405

[33] Antoury L , Hu N , Balaj L , et al. Analysis of extracellular mRNA in human urine reveals splice variant biomarkers of muscular dystrophies. Nat Commun 2018;9:3906. 30254196 10.1038/s41467-018-06206-0 PMC6156576

[34] Das S , Extracellular RNACC , Ansel KM , et al. The Extracellular RNA Communication Consortium: establishing foundational knowledge and technologies for extracellular RNA research. Cell 2019;177:231–242. 30951667 10.1016/j.cell.2019.03.023 PMC6601620

[35] Cheng C , Fass DM , Folz‐Donahue K , MacDonald ME , Haggarty SJ . Highly expandable human iPS cell‐derived neural progenitor cells (NPC) and neurons for central nervous system disease modeling and high‐throughput screening. Curr Protoc Hum Genet 2017;92:2181–2182. 10.1002/cphg.33 PMC5293008 28075486

[36] Guerreiro EM , Vestad B , Steffensen LA , et al. Efficient extracellular vesicle isolation by combining cell media modifications, ultrafiltration, and size‐exclusion chromatography. PLoS ONE 2018;13:e0204276. 30260987 10.1371/journal.pone.0204276 PMC6160036

[37] Vidal M . Exosomes: revisiting their role as “garbage bags”. Traffic 2019;20:815–828. 31418976 10.1111/tra.12687

[38] Janas T , Janas MM , Sapoń K , Janas T . Mechanisms of RNA loading into exosomes. FEBS Lett 2015;589:1391–1398. 25937124 10.1016/j.febslet.2015.04.036

[39] Ragusa M , Barbagallo C , Cirnigliaro M , et al. Asymmetric RNA distribution among cells and their secreted exosomes: biomedical meaning and considerations on diagnostic applications. Front Mol Biosci 2017;4:66. 29046875 10.3389/fmolb.2017.00066 PMC5632685

[40] O'Brien K , Breyne K , Ughetto S , Laurent LC , Breakefield XO . RNA delivery by extracellular vesicles in mammalian cells and its applications. 10.1038/s41580-020-0251-y PMC7249041 32457507

[41] Abels ER , Breakefield XO . Introduction to extracellular vesicles: biogenesis, RNA cargo selection, content, release, and uptake. Cell Mol Neurobiol 2016;36:301–312. 27053351 10.1007/s10571-016-0366-z PMC5546313

[42] Rajendran L , Honsho M , Zahn TR , et al. Alzheimer's disease β‐amyloid peptides are released in association with exosomes. Proc Natl Acad Sci U S A 2006;103:11172–11177. 16837572 10.1073/pnas.0603838103 PMC1544060

[43] Asai H , Ikezu S , Tsunoda S , et al. Depletion of microglia and inhibition of exosome synthesis halt tau propagation. Nat Neurosci 2015;18:1584–1593. 26436904 10.1038/nn.4132 PMC4694577

[44] Silverman JM , Fernando SM , Grad LI , et al. Disease mechanisms in ALS: misfolded SOD1 transferred through exosome‐dependent and exosome‐independent pathways. Cell Mol Neurobiol 2016;36:377–381. 26908139 10.1007/s10571-015-0294-3 PMC11482315

[45] Taylor DD , Gercel‐Taylor C . Exosome platform for diagnosis and monitoring of traumatic brain injury. Philos Trans R Soc B Biol Sci 2014;369:20130503. 10.1098/rstb.2013.0503 PMC4142024 25135964

[46] Wei Z , Batagov AO , Schinelli S , et al. Coding and noncoding landscape of extracellular RNA released by human glioma stem cells. Nat Commun. 2017;8:1–15. 29074968 10.1038/s41467-017-01196-x PMC5658400

[47] Liu W , Bai X , Zhang A , Huang J , Xu S , Zhang J . Role of exosomes in central nervous system diseases. Front Mol Neurosci 2019;12:240. 31636538 10.3389/fnmol.2019.00240 PMC6787718

[48] Otake K , Kamiguchi H , Hirozane Y . Identification of biomarkers for amyotrophic lateral sclerosis by comprehensive analysis of exosomal mRNAs in human cerebrospinal fluid. BMC Med Genom 2019;12:7. 10.1186/s12920-019-0473-z PMC6329125 30630471

[49] Fiandaca MS , Kapogiannis D , Mapstone M , et al. Identification of preclinical Alzheimer's disease by a profile of pathogenic proteins in neurally derived blood exosomes: a case‐control study. Alzheimer's Dement 2015;11:600–607.e1. 25130657 10.1016/j.jalz.2014.06.008 PMC4329112

[50] Zhang X , Abels ER , Redzic JS , Margulis J , Finkbeiner S , Breakefield XO . Potential transfer of polyglutamine and CAG‐repeat RNA in extracellular vesicles in huntington's disease: background and evaluation in cell culture. Cell Mol Neurobiol 2016;36:459–470. 26951563 10.1007/s10571-016-0350-7 PMC5844350

[51] Evidente VGH , Nolte D , Niemann S , et al. Phenotypic and molecular analyses of X‐linked dystonia‐parkinsonism (“lubag”) in women. Arch Neurol 2004;61:1956–1959. 15596620 10.1001/archneur.61.12.1956

[52] Domingo A , Lee LV , Brüggemann N , et al. Woman with X‐linked recessive dystonia‐parkinsonism: clue to the epidemiology of parkinsonism in Filipino women? JAMA Neurol 2014;71:1177–1180. 25004170 10.1001/jamaneurol.2014.56

[53] Domingo A , Westenberger A , Lee LV , et al. New insights into the genetics of X‐linked dystonia‐parkinsonism (XDP, DYT3). Eur J Hum Genet 2015;23:1334–1340. 25604858 10.1038/ejhg.2014.292 PMC4592086

[54] Kawarai T , Morigaki R , Kaji R , Goto S . Clinicopathological phenotype and genetics of X‐linked dystonia‐parkinsonism (XDP; DYT3; Lubag). Brain Sci 2017;7:72. 10.3390/brainsci7070072 PMC5532585 28672841

[55] O'Rawe JA , Wu Y , Dörfel MJ , et al. TAF1 variants are associated with dysmorphic features, intellectual disability, and neurological manifestations. Am J Hum Genet 2015;97:922–932. 26637982 10.1016/j.ajhg.2015.11.005 PMC4678794

[56] Hurst SE , Liktor‐Busa E , Moutal A , et al. A novel variant in TAF1 affects gene expression and is associated with X‐linked TAF1 intellectual disability syndrome. Neuronal Signal 2018;2:NS20180141. 32714589 10.1042/NS20180141 PMC7373232

[57] Gudmundsson S , Wilbe M , Filipek‐Górniok B , et al. TAF1, associated with intellectual disability in humans, is essential for embryogenesis and regulates neurodevelopmental processes in zebrafish. Sci Rep 2019;9:1–11. 31341187 10.1038/s41598-019-46632-8 PMC6656882

