# Supplementary figures and images for: TAF1 Transcripts and Neurofilament Light Chain as Biomarkers for X‐linked Dystonia‐Parkinsonism
Source: Mov Disord. 2020 Sep 25;36(1):206–15. doi: 10.1002/mds.28305 (PMC7891430; doi:10.1002/mds.28305)

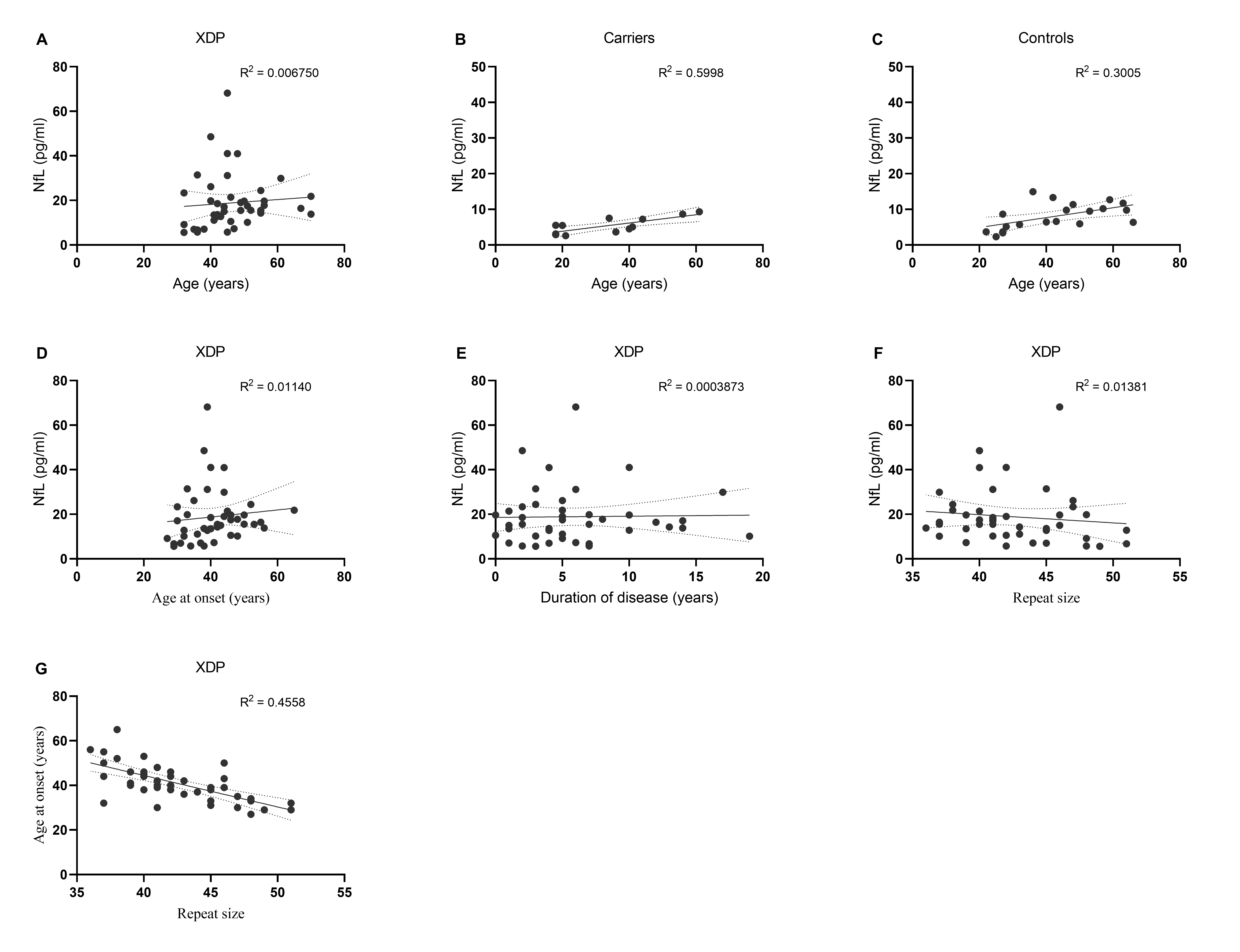

Supplement: Supplementary file 1 — Figure S1. Correlation analysis between neurofilament light chain (NfL) in plasma and disease parameters. (A) Linear correlation between plasma NfL and age in patients with X‐linked dystonia‐parkinsonism (XDP). (B) Linear correlation between plasma NfL and age in asymptomatic female carriers. (C) Linear correlation between plasma NfL and age in asymptomatic controls. (D) Linear correlation between plasma NfL and age at onset of disease in patients with XDP. (E) Linear correlation between plasma NfL and duration of disease in patients with XDP. (F) Linear correlation between plasma NfL and hexanucleotide repeat size in patients with XDP. (G) Linear correlation between plasma age at onset of disease and hexanucleotide repeat size in patients with XDP. [file MDS-36-206-s001.tif]
