# Supplementary material for: TAF1 Transcripts and Neurofilament Light Chain as Biomarkers for X‐linked Dystonia‐Parkinsonism
Source: Mov Disord. 2020 Sep 25;36(1):206–15. doi: 10.1002/mds.28305 (PMC7891430; doi:10.1002/mds.28305)
Supplement: Supplementary file 2 — Table S1. Patient characteristics and results. [file MDS-36-206-s002.docx]

| **Clinical Status** | **Sex** | **Age** | **Repeat Size** | **IInitial Symptom** | **Age at onset** | **Duration of disease** | **TAF1-3'/5'** | **TAF1-32i** | **NfL** | **NfL CV** |
| --- | --- | --- | --- | --- | --- | --- | --- | --- | --- | --- |
| XDP^1,2,3^ | M | 32 | 39 | Dystonia | 29 | 3 | 0.759 | 117.70 | 5.60 | 5.01 |
| XDP^1,2,3^ | M | 45 | 42 | Dystonia | 38 | 7 | 0.702 | 177.93 | 5.77 | 4.23 |
| XDP^1,2,3^ | M | 36 | 48 | Dystonia | 34 | 2 | 0.746 | 148.65 | 5.77 | 4.42 |
| XDP^1,2,3^ | M | 36 | 51 | Dystonia | 29 | 7 | 0.826 | 2876.46 | 6.77 | 0.80 |
| XDP^1,2,3^ | M | 35 | 45 | Dystonia | 31 | 4 | 0.779 | 5552.23 | 7.04 | 9.22 |
| XDP^1,2,3^ | M | 38 | 42 | Dystonia | 37 | 1 | 0.698 | 166.11 | 7.08 | 4.52 |
| XDP^1,2,3^ | M | 47 | 39 | Dystonia | 41 | 6 | 0.725 | 498.74 | 7.27 | 3.12 |
| XDP^1,2,3^ | M | 51 | 37 | Dystonia | 32 | 19 | 0.811 | 117.59 | 10.15 | 2.34 |
| XDP^1,2,3^ | M | 43 | 45 | Dystonia | 39 | 4 | 0.798 | 559.92 | 12.75 | 5.98 |
| XDP^1,2,3^ | M | 41 | 39 | Dystonia | 40 | 1 | 0.671 | 1430.50 | 13.48 | 2.60 |
| XDP^1,2,3^ | M | 55 | 43 | Dystonia | 42 | 13 | 0.745 | 127.34 | 14.30 | 3.64 |
| XDP^1,2,3^ | M | 44 | 46 | Dystonia | 43 | 1 | 0.760 | 154.02 | 15.05 | 1.86 |
| XDP^1,2,3^ | M | 55 | 44 | Dystonia | 53 | 2 | 0.745 | 0.16 | 15.49 | 0.61 |
| XDP^1,2,3^ | M | 49 | 41 | Dystonia | 42 | 7 | 0.627 | 21.70 | 15.51 | 0.83 |
| XDP^1,2^ | M | 53 | 37 | Dystonia | 50 | 3 | 0.673 | 31.33 | 15.57 | 5.28 |
| XDP^1,2,3^ | M | 44 | 41 | Dystonia | 30 | 14 | 0.767 | 1877.29 | 17.08 | 6.60 |
| XDP^1,2,3^ | M | 51 | 40 | Dystonia | 46 | 5 | 0.738 | 1.47 | 17.53 | 4.95 |
| XDP^1,2,3^ | M | 55 | 41 | Dystonia | 48 | 7 | 0.677 | 189.02 | 17.74 | 4.83 |
| XDP^1,2,3^ | M | 49 | 42 | Dystonia | 44 | 5 | 0.610 | 53.50 | 19.05 | 0.00 |
| XDP^1,2,3^ | M | 40 | 48 | Dystonia | 33 | 7 | 0.653 | 48.89 | 19.83 | 1.40 |
| XDP^1,2,3^ | M | 46 | 40 | Dystonia | 45 | 1 | 0.698 | 624.94 | 21.42 | 5.23 |
| XDP^1,2,3^ | M | 70 | 38 | Dystonia | 65 | 5 | 0.753 | 105.18 | 21.81 | 3.16 |
| XDP^1,2,3^ | M | 32 | 47 | Dystonia | 30 | 2 | 0.679 | 109.28 | 23.37 | 0.83 |
| XDP^1,2,3^ | M | 55 | 38 | Dystonia | 52 | 3 | 0.641 | 0.72 | 24.44 | 0.88 |
| XDP^1,2,3^ | M | 36 | 45 | Dystonia | 33 | 3 | 0.595 | 102.05 | 31.46 | 3.42 |
| XDP^1,2,3^ | M | 48 | 40 | Dystonia | 44 | 4 | 0.622 | 0.34 | 40.95 | 3.07 |
| XDP^1,2,3^ | M | 40 | 40 | Dystonia | 38 | 2 | 0.717 | 1195.58 | 48.56 | 0.92 |
| XDP^1,2,3^ | M | 45 | 46 | Dystonia | 39 | 6 | 0.745 | 0.48 | 68.19 | 6.54 |
| XDP^1,2^ | M | 85 | 41 | Dystonia | 48 | 37 | 0.709 | 1038.83 | - | - |
| XDP^1,2^ | M | 54 | 42 | Dystonia | 41 | 13 | 0.539 | 570.53 | - | - |
| XDP^1,3^ | M | 45 | 42 | Dystonia | 35 | 10 | 0.713 | - | 41.02 | 0.99 |
| XDP^1,3^ | M | 40 | 47 | Dystonia | 35 | 5 | 0.691 | - | 26.19 | 2.24 |
| XDP^1,3^ | M | 51 | 41 | Dystonia | 48 | 3 | 0.634 | - | 10.26 | 1.00 |
| XDP^1,3^ | M | 45 | 41 | Dystonia | 39 | 6 | 0.746 | - | 31.16 | 4.00 |
| XDP^2,3^ | M | 56 | 39 | Dystonia | 46 | 10 | - | 223.53 | 19.69 | 2.54 |
| XDP^3^ | M | 70 | 36 | Dystonia | 56 | 14 | - | - | 13.86 | 5.91 |
| XDP^3^ | M | 42 | 45 | Dystonia | 38 | 4 | - | - | 13.60 | 6.87 |
| XDP^3^ | M | 42 | 51 | Dystonia | 32 | 10 | - | - | 12.84 | 1.15 |
| XDP^1,2,3^ | M | 67 | 37 | Parkinsonism | 55 | 12 | 0.888 | 1.21 | 16.42 | 7.60 |
| XDP^1,2,3^ | M | 46 | 45 | Parkinsonism | 46 | 0 | 0.818 | 1574.28 | 10.56 | 1.33 |
| XDP^1,2,3^ | M | 41 | 43 | Parkinsonism | 36 | 5 | 0.556 | 209.22 | 11.09 | 2.00 |
| XDP^1,2,3^ | M | 61 | 37 | Parkinsonism | 44 | 17 | 0.737 | 725.28 | 29.87 | 9.38 |
| XDP^1,2,3^ | M | 32 | 48 | Parkinsonism | 27 | 5 | 0.745 | 63.65 | 9.17 | 0.67 |
| XDP^1,2,3^ | M | 42 | 41 | Parkinsonism | 40 | 2 | 0.646 | 126.43 | 18.56 | 4.86 |
| XDP^1,2,3^ | M | 50 | 46 | Parkinsonism | 50 | 0 | 0.762 | 0.65 | 19.70 | 1.92 |
| XDP^1,2^ | M | 59 | 43 | Parkinsonism | 37 | 22 | 0.638 | 0.80 | - | - |
| XDP^1,2^ | M | 54 | 37 | Parkinsonism | 43 | 11 | 0.753 | 1001.72 | - | - |
| XDP^1,2,3^ | M | 29 | 51 | Pre-symptomatic | na | na | 0.738 | 23.99 | 3.48 | 1.89 |
| XDP^1,3^ | M | 18 | 46 | Pre-symptomatic | na | na | 0.750 | - | 5.50 | 3.54 |
| XDP^1,2^ | M | 51 | 36 | Pre-symptomatic | na | na | 0.814 | 2320.66 | - | - |
|  |  |  |  |  |  |  |  |  |  |  |

**Supplementary Table S1: Patient subjects characteristics and results.**

XDP patient samples used for TAF1 expression analysis assays and plasma NfL assay. TAF1-3’/5’ ratio, TAF1-32i expression, and mean plasma NfL concentration (pg/ml) were reported for each sample. ^1^Samples used for TAF1-3’/5’ expression analysis, ^2^Samples used for TAF1-32i expression analysis, ^3^Samples used for plasma NfL analysis. CV: coefficients of variation.
