# Supplementary material for: TAF1 Transcripts and Neurofilament Light Chain as Biomarkers for X‐linked Dystonia‐Parkinsonism
Source: Mov Disord. 2020 Sep 25;36(1):206–15. doi: 10.1002/mds.28305 (PMC7891430; doi:10.1002/mds.28305)
Supplement: Supplementary file 3 — Table S2. Carrier and control characteristics and results. [file MDS-36-206-s003.docx]

| **Clinical Status** | **Sex** | **Age** | **Symptoms/Signs of disease** | **TAF1-3'/5'** | **TAF1-32i** | **NfL** | **NfL CV** |
| --- | --- | --- | --- | --- | --- | --- | --- |
| Carrier^1,2^ | F | 84 | - | 1.046 | 601.30 | - | - |
| Carrier^1,2^ | F | 58 | - | 0.762 | 1.60 | - | - |
| Carrier^1,2^ | F | 27 | - | 1.109 | 411.34 | - | - |
| Carrier^1,2^ | F | 47 | - | 0.904 | 883.88 | - | - |
| Carrier^1,2,3^ | F | 36 | - | 0.781 | 1511.18 | 3.63 | 21.65 |
| Carrier^1,2,3^ | F | 18 | - | 0.902 | 8.65 | 5.52 | 5.42 |
| Carrier^1,2,3^ | F | 41 | - | 0.965 | 0.00 | 5.06 | 6.72 |
| Carrier^1,2,3^ | F | 56 | - | 0.846 | 8.49 | 8.64 | 5.52 |
| Carrier^1,2,3^ | F | 44 | - | 0.975 | 23.99 | 7.25 | 4.48 |
| Carrier^1,2,3^ | F | 40 | - | 0.837 | 834.29 | 4.51 | 7.19 |
| Carrier^1,2,3^ | F | 18 | - | 0.991 | 681.10 | 2.91 | 10.65 |
| Carrier^1,2,3^ | F | 20 | - | 0.856 | 305.90 | 5.46 | 1.79 |
| Carrier^1,2,3^ | F | 21 | - | 0.827 | 0.77 | 2.61 | 0.18 |
| Carrier^3^ | F | 18 | - | - | - | 3.11 | 4.39 |
| Carrier^3^ | F | 61 | - | - | - | 9.32 | 1.93 |
| Carrier^3^ | F | 34 | - | - | - | 7.51 | 2.44 |
| Carrier^1,2,3^ | F | 84 | Parkinsonism | 0.880 | 116.24 | 71.99 | 5.33 |
| Carrier^1,2,3^ | F | 77 | Parkinsonism | 0.792 | 4.56 | 14.10 | 7.05 |
| Carrier^1,2,3^ | F | 77 | Parkinsonism | 0.842 | 419.04 | 26.85 | 4.74 |
| Carrier^1,2,3^ | F | 78 | Parkinsonism | 0.981 | 1457.98 | 87.67 | 4.15 |
| Control^1,2,3^ | F | 59 | - | 1.091 | 1.21 | 12.67 | 5.38 |
| Control^1,2,3^ | M | 27 | - | 1.083 | 0.45 | 8.66 | 6.85 |
| Control^1,2,3^ | M | 27 | - | 1.099 | 0.15 | 3.67 | 4.13 |
| Control^1,2,3^ | F | 66 | - | 1.034 | 0.13 | 6.37 | 2.74 |
| Control^1,2,3^ | M | 36 | - | 1.027 | 0.00 | 14.93 | 0.85 |
| Control^1,2,3^ | M | 46 | - | 0.897 | 7.63 | 9.76 | 9.36 |
| Control^1,2,3^ | F | 58 | - | 1.087 | 0.00 | 5.96 | 2.89 |
| Control^1,2,3^ | M | 42 | - | 1.062 | 0.00 | 13.31 | 4.36 |
| Control^1,2,3^ | M | 27 | - | 0.957 | 0.00 | 5.13 | 2.95 |
| Control^1,2,3^ | F | 64 | - | 0.879 | 0.12 | 9.79 | 2.75 |
| Control^1,2^ | F | 51 | - | 0.878 | 0.15 | - | - |
| Control^1,2^ | F | 26 | - | 1.048 | 0.00 | - | - |
| Control^1,2^ | F | 53 | - | 1.071 | 1.30 | - | - |
| Control^1,2^ | M | 20 | - | 0.890 | 1.09 | - | - |
| Control^1,2^ | M | 28 | - | 0.957 | 5.39 | - | - |
| Control^1,2^ | M | 36 | - | 0.971 | 0.39 | - | - |
| Control^3^ | M | 57 | - | - | - | 10.20 | 1.39 |
| Control^3^ | F | 63 | - | - | - | 11.73 | 3.51 |
| Control^3^ | M | 43 | - | - | - | 6.59 | 11.22 |
| Control^3^ | M | 49 | - | - | - | 114.44 | 0.45 |
| Control^3^ | M | 27 | - | - | - | 3.41 | 12.35 |
| Control^3^ | M | 22 | - | - | - | 3.69 | 10.24 |
| Control^3^ | F | 25 | - | - | - | 2.32 | 26.92 |
| Control^3^ | M | 40 | - | - | - | 6.42 | 2.62 |
| Control^3^ | M | 53 | Parkinsonism | - | - | 9.47 | 1.70 |
| Control^1,2,3^ | M | 32 | Parkinsonism | 1.080 | 0.00 | 5.73 | 5.28 |
| Control^1,2,3^ | M | 48 | Parkinsonism | 0.926 | 0.00 | 11.34 | 0.23 |
|  |  |  |  |  |  |  |  |

**Supplementary Table S2: Carrier and control subjects characteristics and results.**

Carrier and control samples used for TAF1 expression analysis assays and plasma NfL assay. TAF1-3’/5’ ratio, TAF1-32i expression, and mean plasma NfL concentration (pg/ml) were reported for each sample. ^1^Samples used for TAF1-3’/5’ expression analysis, ^2^Samples used for TAF1-32i expression analysis, ^3^Samples used for plasma NfL analysis. CV: coefficients of variation.
